# 324. The Impact of Adding Staff-performed Telephone Call Reminders to Automated Call Reminders in An Infectious Diseases Outpatient Practice

**DOI:** 10.1093/ofid/ofad500.395

**Published:** 2023-11-27

**Authors:** Mery Deeb, Zainah Hadeel

**Affiliations:** Brown University/Kent Hospital, warwick, Rhode Island; Kent-County Memorial Hospital, Warwick, Rhode Island

## Abstract

**Background:**

Automated text message service reminders have been shown to increase show-up rates in outpatient practices. Furthermore, automated telephone call service reminders also decrease the no-show rates; however, no-show rates have decreased even further when telephone call reminders were done by health-care personnel. We sought to demonstrate the benefit of telephone call reminders done by clinic staff when added to the automated call reminders on improving show-up rates.

**Methods:**

This was a quality improvement project, which was conducted by performing prospective data collection from our outpatient Infectious Diseases (ID) practice between May 2021 and May 2022. The outpatient ID practice served a 359-bed community hospital. There were two providers in the ID practice. The automated call reminder system was utilized for all patients’ visits. Starting November 2021, our medical assistant performed a telephone call reminder one day prior to the visit. We compared the rates of no-shows between May 2021 to November 2021 to those between November 2021 and May 2022

**Results:**

In a period of 6 months, there were 277 patients scheduled for outpatient visits. The automated call reminders were being utilized routinely. There were 18 patients (7.93%) who did not present to the scheduled appointments. In the following 7 months, after incorporating staff reminder calls, 21 of 321 scheduled patients (6.54%) did not show up to the visits. There was a decrease of 1.39 % in the no-show rate after the staff call implementation (P-value of 1). The No-show rate was different between the two providers. No-show rate decreased from 6.76% to 5.68% for provider A (P-value: 0.8), compared to a decrease from 10.13% to 7.59% for provider B (P-value: 0.6).

**Conclusion:**

When done one day prior to the appointment, staff-performed call reminders did not improve show-up rates when added to the automated call reminders in the Infectious Diseases outpatient practice. Further studies are needed to evaluate the impact of adding staff-performed call reminders at a different interval from the scheduled appointments.

**Disclosures:**

**All Authors**: No reported disclosures

